# Emergence of waterfowl‐originated gene cassettes in HPAI H7N9 viruses caused severe human infection in Fujian, China

**DOI:** 10.1111/irv.12657

**Published:** 2019-06-11

**Authors:** Lei Yang, Jianfeng Xie, Ye Zhang, Wenfei Zhu, Xiyan Li, Hejiang Wei, Zi Li, Lin Zhao, Hong Bo, Jia Liu, Jie Dong, Tao Chen, Yuelong Shu, Yuwei Weng, Dayan Wang

**Affiliations:** ^1^ National Institute for Viral Disease Control and Prevention Chinese Center for Disease Control and Prevention Beijing China; ^2^ Fujian center for disease control and prevention Fuzhou China; ^3^ Fujian provincial key laboratory of zoonosis research Fuzhou China; ^4^ School of Public Health Shenzhen Sun Yat‐sen University Guangdong China

**Keywords:** avian influenza, genetic diversity, H7N9 virus, infection

## Abstract

**Background:**

Highly pathogenic avian influenza (HPAI) A(H7N9) virus emerged and caused human infections during the 2016‐2017 epidemic wave of influenza A(H7N9) viruses in China. We report a human infection with HPAI H7N9 virus and six environmental isolates in Fujian Province, China.

**Methods:**

Environmental surveillance was conducted in live poultry markets and poultry farms in Fujian, China. Clinical and epidemiologic data and samples were collected. Real‐time RT‐PCRs were conducted for each sample, and H7‐positive samples were isolated using embryonated chicken eggs. Full genomes of the isolates were obtained by next‐generation sequencing. Phylogenetic analysis and antigenic analysis were conducted.

**Results:**

A 59‐year‐old man who raised about 1000 ducks was identified as HPAI H7N9 infection. Six HPAI H7 viruses were isolated from environmental samples, including five H7N9 viruses and one H7N6 virus. Phylogenetic results showed the human and environmental viruses are highly genetically diverse and containing significantly different gene constellation from that of other HPAI H7N9 previously reported. The internal genes derived from H7N9/H9N2, H5N6, and the Eurasian wild‐bird gene pool, indicating waterfowl‐originated genotypes, have emerged in HPAI H7N9/N6 viruses and caused human infection.

**Conclusion:**

The new genotypes raise the concern that these HPAI H7 viruses might transmit back into migratory birds and spread to other countries as the HPAI H5Nx viruses. Considering their capability of causing severe infections in both human and poultry, the HPAI H7 viruses in this study pose a risk to public health and the poultry industry and highlight the importance of sustained surveillance of these viruses.

## 1 INTRODUCTION

Avian influenza viruses (AIV) belong to type A influenza virus, whose genome consists of eight segments of single‐stranded RNA. The segments 4 and 6 encode two surface proteins, the hemagglutinin (HA) and neuraminidase (NA), which could subtype AIVs into H1‐H16 and N1‐N9, respectively. Other six segments encode the viral internal genes: polymerase basic protein 2 (PB2, segment 1), polymerase basic protein 1 (PB1, segment 2), polymerase acidic protein (PA, segment 3), nucleocapsid protein (NP, segment 5), the matrix proteins (MP, segment 7), and the nonstructural proteins (NS, segment 8). Reassortment of segments originated from different hosts may facilitate virus cross‐species transmission.

Since 2013, the three original reassortment low pathogenic avian influenza (LPAI) A(H7N9) viruses have emerged and caused five epidemic waves with over 1500 human cases in China.^1^ In early 2017, human infections with a highly pathogenic avian influenza (HPAI) A(H7N9) virus were reported in Taiwan and Guangdong Provinces of China.^2^, ^3^ Phylogenic analyses showed that the HPAI H7N9 virus originated from the LPAI H7N9 virus.^4^, ^5^ The signature genetic difference between HPAI and LPAI H7N9 virus was an insertion of four amino acids at the cleavage site of the HA protein.^3^, ^6^ Biological assays both in vitro and in vivo indicated that this insertion caused a switch in the virulence of the H7N9 virus in poultry.^6^, ^7^, ^8^

To date, most evidence supports the notion that the insertion event probably occurred in poultry in the Pearl River Delta region and that the Guangdong Province was the original location of the HPAI H7N9 virus.^4^, ^5^ The HPAI H7N9 virus might have emerged in mid‐2016, according to a molecular clock model.^4^, ^5^ Frequent reassortments of internal genes among H7N9 and H9N2 viruses have been observed in the HPAI H7N9 viruses, similar to those in the LPAI H7N9 viruses.^4^, ^5^, ^9^ As of December 19, 2018, twenty‐seven outbreaks in poultry have been reported in 12 provinces in China, about 900 000 poultry have been culled (http://www.oie.int). And 32 human cases have been reported, with the fatality rate of 43.8% (14/32) (http://www.chinaivdc.cn/cnic/).

Fujian Province borders the northeast part of Guangdong Province and the southern part of Zhejiang Province. Both Guangdong and Zhejiang Provinces are located in the outbreak sources (Pearl River Delta region and Yangtze River Delta region, respectively) of the LPAI H7N9 viruses.^10^ The introduction of LPAI H7N9 viruses from both sources into Fujian Province has been documented,^10^ and these have caused continuous human infections since the spring of 2013. Here, we report a human case infected with HPAI H7N9 in Fujian Province, in August 2017. Genetic studies of the human and environmental isolates have shown that these HPAI H7 viruses are highly genetically diverse, with internal genes from wild‐bird viruses.

## 2 MATERIALS AND METHODS

### 2.1 Ethics statement

As a public health response to the outbreak, written informed consent of the study subjects could be waived according to Chinese law. The clinical sample collection and transportation were performed according to the Chinese Guidelines for the Diagnosis and Treatment of Human Infection with H7N9 Avian Influenza Virus (2nd edition, 2013).

### 2.2 Clinical and epidemiologic data collection

The clinical history and epidemiologic information were obtained from epidemiological investigation reports, conducted by local centers for disease control and prevention.

### 2.3 Sample collection and isolation

Respiratory specimens from patients and environmental samples including swabs of cages, feces, chopping boards, sewage, and poultry drinking water from live poultry markets or farms were collected by local centers for disease control and prevention, according to the *national influenza surveillance guidelines*. Each sample was collected in individual vials, placed in transport medium with antibiotics, and packed on ice before it was sent to the laboratory for further processing.

Real‐time reverse transcription‐PCRs (real‐time RT‐PCRs) were conducted to detect the influenza A virus and the H7 subtype in the original samples. Viral RNA was extracted (QIAamp viral RNA mini kit, Qiagen, Germany). The primers and probe were as follows: FluA‐Forward 5′‐GAC CRA TCC TGT CAC CTC TGA C‐3′, FluA‐Reverse 5′‐GGG CAT TYT GGA CAA AKC GTC TAC G‐3′, FluA‐probe 5′‐TGC AGT CCT CGC TCA CTG GGC ACG −3′, H7‐forward (5′‐AGA AAT GAA ATG GCT CCT GTC AA‐3′), H7‐reverse (5′‐GGT TTT TTC TTG TAT TTT TAT ATG ACT TAG‐3′), and H7‐probe (5′‐AGA TAA TGC TGC ATT CCC GCA GAT G‐3′). RNAs were amplified using one‐step RT‐PCR (OneStep RT‐PCR Kit, Qiagen, Germany). Reactions, detection, and data analyses were performed with Applied Biosystems^TM ^real‐time PCR systems 7500.

To isolate the virus, 9‐ to 11‐day‐old specific‐pathogen‐free embryonated chicken eggs were inoculated with H7‐positive samples for 48‐72 hours at 37°C in BSL‐3 laboratory. The allantoic fluid was harvested. And the viral titer was determined by hemagglutination assay. The positive fluid was stored at −80°C.

### 2.4 Sequencing

RNA was extracted from each positive isolate with the RNeasy Mini Kit (Qiagen, Hilden, Germany). The extracted RNA was subjected to reverse transcription and amplification with the SuperScript III One‐Step RT‐PCR System (Thermo Fisher, Waltham, MA, USA) using primers (5′‐GGGGGGAGCAAAAGCAGG‐3′, 5′‐GGGGGGAGCGAAAGCAGG‐3′ and 5′‐CGGGTTATTAGTAGAAACAAGG‐3′). The whole genomes of influenza A(H7N9) virus were sequenced on the MiSeq high‐throughput sequencing platform (Illumina, Inc, San Diego, CA, USA) with a paired read length of 150 bp. We predominantly analyzed the data with the CLC Genomics Workbench 7.5.1 software. The raw data were trimmed and assembled de novo. Contigs were compared with BLAST against the Global Initiative on Sharing All Influenza Data (GISAID) database. Sequences with the highest similarity were selected as references for read mapping. The genome sequences were obtained by extracting the consensus sequences from the mapping results, with a coverage depth of at least 100 times at each site on the eight segments. The sequences generated in this study were submitted to the GISAID database (accession number: EPI1252096‐EPI1252159).

### 2.5 Phylogenetic analysis

The highly similar sequences detected with BLAST and the publicly available sequences of the Fujian H7N9 viruses (isolated in 2017) were used in a phylogenetic analysis of each gene segment. Multiple alignments were constructed with the MUSCLE tool in the MEGA 5.05 software. Maximum likelihood trees were constructed with RAxML v 8.1.20 using the general time‐reversible plus gamma distribution model. A rapid bootstrap analysis with 1000 replicates was performed for each tree.

## 3 RESULTS

### 3.1 The patient

The patient was a 59‐year‐old man with diabetes who raised about 1000 ducks in Zhangzhou, Fujian Province, China. On July 19, 2017, he suffered a fever and got worse with the highest body temperature 40°C. On July 29, he was admitted to a local hospital and diagnosed as pneumonia. Moxifloxacin and oseltamivir were administered. On July 30, he developed type I respiratory failure and was treated with noninvasive positive pressure mechanical ventilation. On August 1, a sample collected from his alveolar lavage fluid was positive for influenza A/H7N9 virus by real‐time RT‐PCR. The patient's condition improved after August 1. Nasal and throat swabs that were sampled on August 8 and August 10 were all negative. On August 11, the patient recovered and was discharged from the hospital. Full genome sequencing identified the virus isolated from the clinical specimen, A/Fujian/33845/2017(H7N9), as a HPAI H7N9 virus.

This patient lived in his duck farm and had no history of live poultry market visiting within 10 days before his illness onset. No clinical signs were detected in 11 close contacts. Eight of 20 environmental samples collected from his and his neighbor's duck farms were positive for the influenza A/H7 virus by real‐time RT‐PCR, but virus isolation failed.

### 3.2 Environmental surveillance

**Figure 1:**
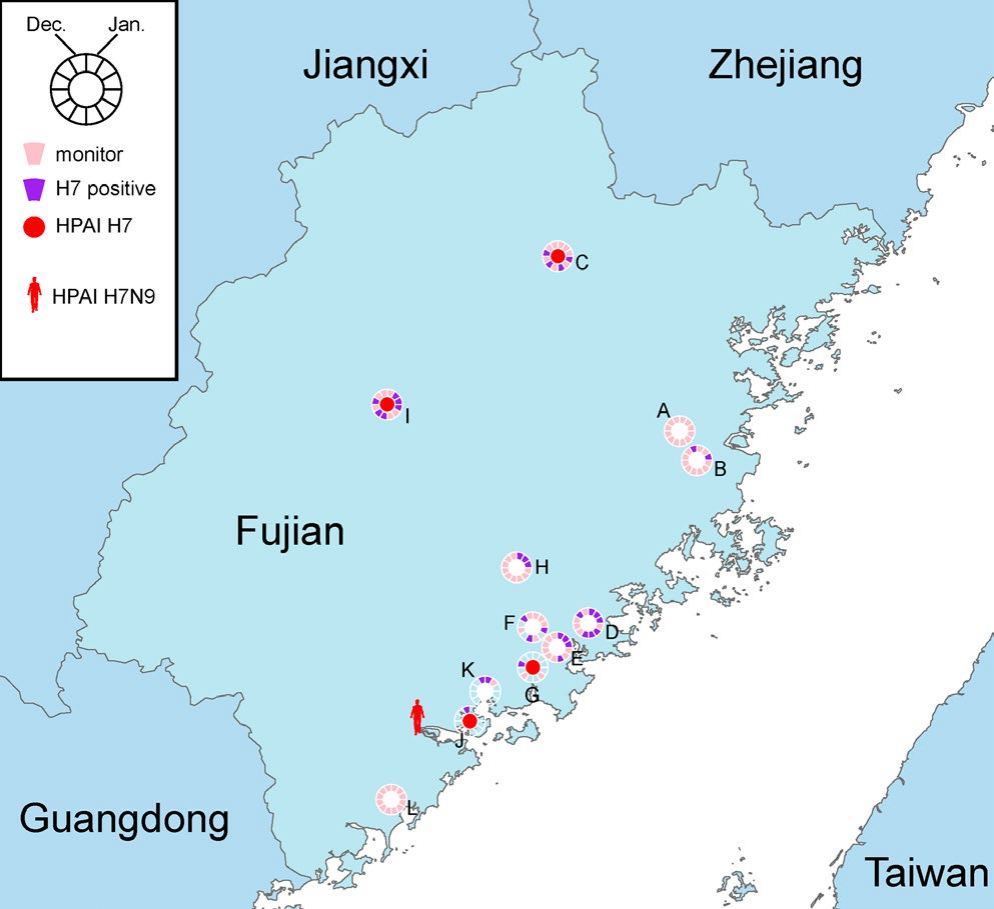
Map of human H7N9 infection and environmental surveillance in Fujian. Location of the human infection is shown in the cartoon. Environmental surveillance sites (A‐L) were labeled with circles, representing January to December. Months in which monitoring was conducted and H7 detected are colored in pink and purple, respectively. Solid red circles indicate where the HPAI H7 virus isolates were sampled

The environmental surveillance on live poultry markets and poultry farms was routinely conducted at 12 surveillance sites (A‐L) in Fujian, China, throughout the year at a monthly manner (Figure 1). A total of 922 samples were collected during 2017. Based on real‐time RT‐PCR assays, 446(45.0%) and 85(9.2%) of them were positive for influenza A and subtype H7, respectively. H7 subtype was detected in Fujian in every month except September, and 10 of the 12 sampling sites were positive for H7 subtype at different time points (Figure 1).

Seven H7 viruses were isolated from the H7‐positive samples. After sequencing, one of them was identified as an LPAI H7N9 virus and six were identified as HPAI H7 viruses including five H7N9 viruses and one H7N6 virus (Table 1). These HPAI H7 viruses were from four surveillance sites and were all collected after August 2017 (Figure 1 and Table 1).

**Table 1:** List of viruses isolated in this study and their molecular characteristics

No.	Strain Name	Subtype	Passage	Sample Date	Sample site	HA^a^			NA^b^	M2	PB2				
						186	226‐228	Cleavage Site of HA	292	31	526	588	591	627	701
1	A/Fujian/33845/2017	H7N9	E1	2017/8/1		V	QSG	PKRKRTAR↓G	R	N	K	A	Q	E	N
2	A/Environment/Fujian/24907/2017	H7N9	E1	2017/3/10	B	V	LSG	P‐‐‐‐KGR↓G	R	N	K	V	Q	E	D
3	A/Environment/Fujian/36998/2017	H7N9	E1	2017/8/14	I	V	QSG	PKRKRTAR↓G	R	N	K	V	Q	E	D
4	A/Environment/Fujian/40791/2017	H7N9	E1	2017/10/18	C	V	QSG	PKRKRAAR↓G	R	N	R	A	Q	E	D
5	A/Environment/Fujian/40844/2017	H7N9	E1	2017/10/24	G	V	QSG	PKRKRTAR↓G	R	N	K	A	Q	E	D
6	A/Environment/Fujian/40843/2017	H7N9	E1	2017/10/24	G	V	QSG	PKRKRTAR↓G	R	N	K	A	Q	E	D
7	A/Environment/Fujian/43639/2017	H7N9	E1	2017/12/5	J	V	QSG	PKRKRTAR↓G	R	N	K	V	Q	E	D
8	A/Environment/Fujian/43658/2017	H7N6	E1	2017/12/6	J	V	QSG	PKRKRTAR↓G	R	N	K	A	Q	E	D

Note:

The sample site was as the same as that shown in Figure 1.

a H3 numbering system was used.

b N2 numbering system was used.

### 3.3 Molecular markers

As shown in Table 1, all HPAI H7 viruses contained a 4‐amino‐acid insertion (“KRTA” or “KRAA”) in the HA cleavage site. The LPAI A/Environment/Fujian/24907/2017(H7N9) virus encoded amino acid residues 186V and 226L (H3 numbering) in the HA protein, while all the HPAI viruses contained 186V and 226Q. Both combinations could recognize avian‐ and human‐type receptors.^7^, ^11^, ^12^ No virus carried the reported NA inhibitor‐resistant mutations, such as E119V, I222R, H274Y, and R292K,^13^ but all contained the amantadine‐resistant S31N mutation in M2 protein. PB2 526R and 588V, which can promote the mammalian adaptation of influenza virus, were detected in four environmental isolates.^14^, ^15^ The mammalian‐adaptive mutation PB2 701N was found in the human isolate.^16^

The amino acids of the HA subunit 1 (HA1) of the HPAI H7 viruses were compared to those of candidate vaccine strain HPAI A/Guangdong/17SF003/2016(H7N9). A total of fourteen mutation sites were observed, and 4‐9 mutations were carried by each isolate (Table 2). The human isolate contained seven mutations including R22K, R47K, G133E, K163R, I169V, R252K, and R261G (H7 HA1 numbering without signal peptide). In these mutations, R47K and G133E have been reported to be resistant to the neutralizing activity of monoclonal antibodies from H7N9‐immunized mouse.^17^ And one and five environmental isolates also have R47K and G133E mutations, respectively.

**Table 2:** Amino acid difference of HA1 compared to candidate vaccine strain A/Guangdong/17SF003/2016

Strains	HA1^a^													
	11	22	47	81	104	115	133	163	165	169	180	196	252	261
A/Guangdong/17SF003/2016(H7N9)	S	R	R	R	E	F	G	K	S	I	A	G	R	R
A/Fujian/33845/2017(H7N9)	.	K	K	.	.	.	E	R	.	V	.	.	K	G
A/Environment/Fujian/36998/2017(H7N9)	P	.	.	.	.	L	.	R	.	.	.	.	.	G
A/Environment/Fujian/40791/2017(H7N9)	.	K	.	Q	.	.	E	R	R	V	.	.	.	G
A/Environment/Fujian/40843/2017(H7N9)	.	K	K	.	.	.	E	R	.	V	.	W	K	G
A/Environment/Fujian/40844/2017(H7N9)	.	K	.	.	.	.	E	R	.	V	.	W	K	G
A/Environment/Fujian/43639/2017(H7N9)	.	K	N	.	G	.	E	R	.	V	E	.	K	G
A/Environment/Fujian/43658/2017(H7N6)	.	K	N	.	G	.	E	R	.	V	E	.	K	G

a The mature H7 numbering without signal peptide system was used. Periods indicate the same amino acid as in A/Guangdong/17SF003/2016(H7N9).

### 3.4 Genetic diversity of H7 viruses

Whole genome sequence comparison between the human isolate and environmental HPAI viruses showed that all 8 segments of the A/Environment/Fujian/40843/2017(H7N9) virus possess the highest identity (average 99.6%, range 99.3‐99.8%) to the human virus, A/Fujian/33845/2017(H7N9) (Table 3). However, other environmental viruses contained at least one segment that had a higher variability compared to the human virus. Higher genetic diversities were observed on these segments: PB2 (86.1‐99.6%), PB1 (89.6‐99.6%), PA (90.6‐99.6%), NP (89.2‐99.3%), NA (64.0‐99.8%), MP (95.1‐99.8%), and NS (69.9‐99.8%).

**Table 3:** The nucleotides identity compared to A/Fujian/33845/2017(H7N9) virus

Strains	PB2	PB1	PA	HA	NP	NA	MP	NS
A/Environment/Fujian/40843/2017(H7N9)	99.6%	99.6%	99.7%	99.6%	99.3%	99.6%	99.8%	99.8%
A/Environment/Fujian/40844/2017(H7N9)	90.7%	99.6%	99.6%	99.6%	99.3%	99.7%	99.8%	99.8%
A/Environment/Fujian/43639/2017(H7N9)	87.8%	99.5%	99.5%	99.5%	89.4%	99.6%	99.1%	99.5%
A/Environment/Fujian/40791/2017(H7N9)	86.4%	89.6%	99.1%	98.8%	99.1%	99.0%	99.1%	99.4%
A/Environment/Fujian/36998/2017(H7N9)	86.1%	89.6%	90.6%	98.6%	89.2%	96.8%	95.1%	69.9%
A/Environment/Fujian/43658/2017(H7N6)	99.6%	99.5%	99.5%	99.6%	89.7%	64.0%	99.3%	99.5%

### 3.5 Origins of eight segments of HPAI H7 viruses

**Figure 2:**
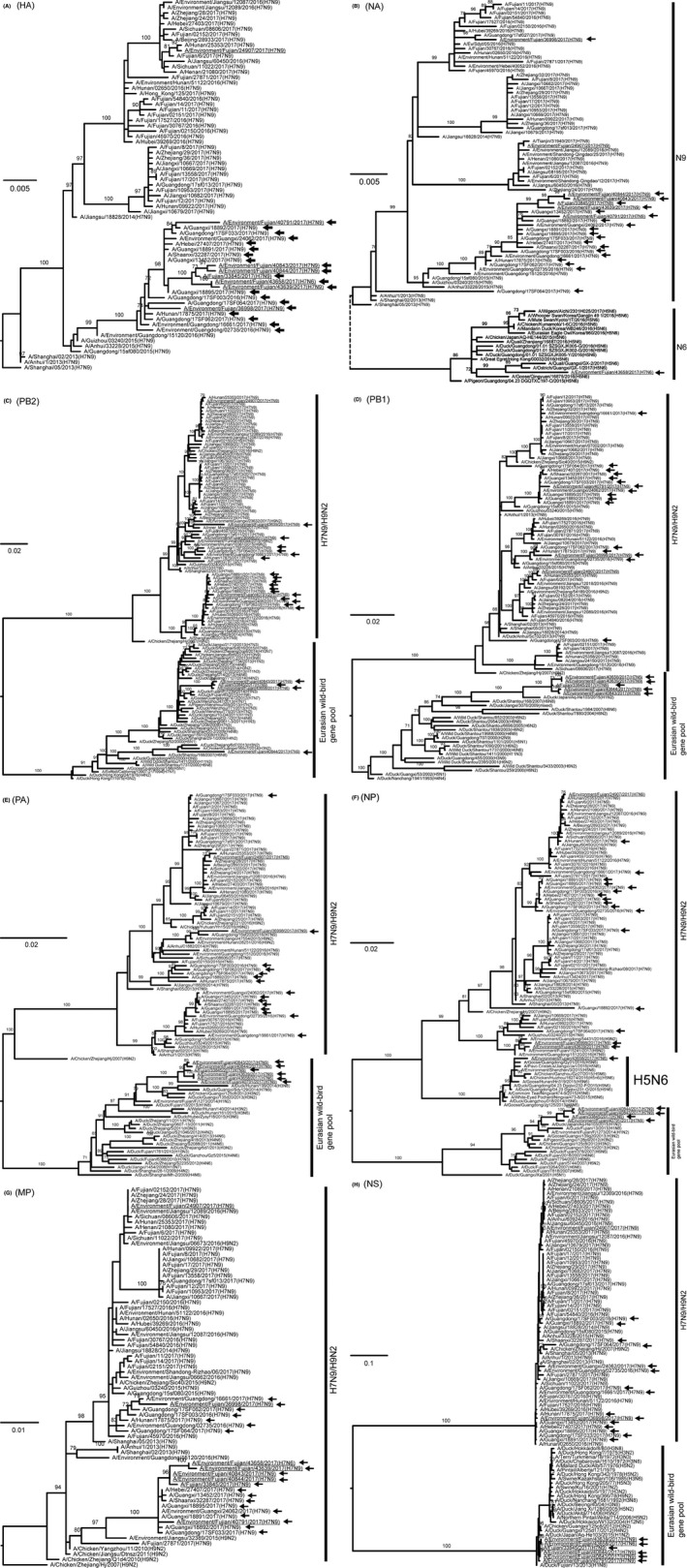
Maximum likelihood trees of HA (A), NA (B), PB2 (C), PB1 (D), PA (E), NP (F), MP (G), and NS (H) of the influenza A/H7 viruses. The viruses identified in this study are underlined. The HPAI H7 viruses are pointed by arrows. Only bootstrap values greater than 70 are shown

To identify the origins of the genetic diversity, phylogenetic analyses were conducted for all eight segments with sequences from the GISAID EpiFlu™ database, which have a higher similarity with viruses in this study, by BLAST (Figure 2). The HA genes of the human isolate and six environmental HPAI H7 isolates clustered with those of other HPAI H7N9 viruses (Figure 2A) and had the same origin as those of the HPAI H7N9 viruses previously reported,^4^, ^5^ while the NA gene of one of the viruses, A/Environment/Fujian/43658/2017(H7N6), was from H5N6 virus (Figure 2B).

Previous studies showed that the internal genes of the HPAI H7N9 viruses were derived from H7N9/H9N2 viruses^4^, ^9^; however, the internal genes of these HPAI H7 viruses, including the human virus, have three sources, H7N9/H9N2 from poultry, H5N6, and the Eurasian wild‐bird gene pool (Figure 2C‐H). PB2, PB1, PA, and NS genes were derived from H7N9/H9N2 or the Eurasian wild‐bird gene pool, while the NP gene was from H7N9/H9N2 from poultry, H5N6, or the Eurasian wild‐bird gene pool. The MP genes of these isolates were all closely related to the other LPAI and HPAI H7N9 viruses.

**Figure 3:**
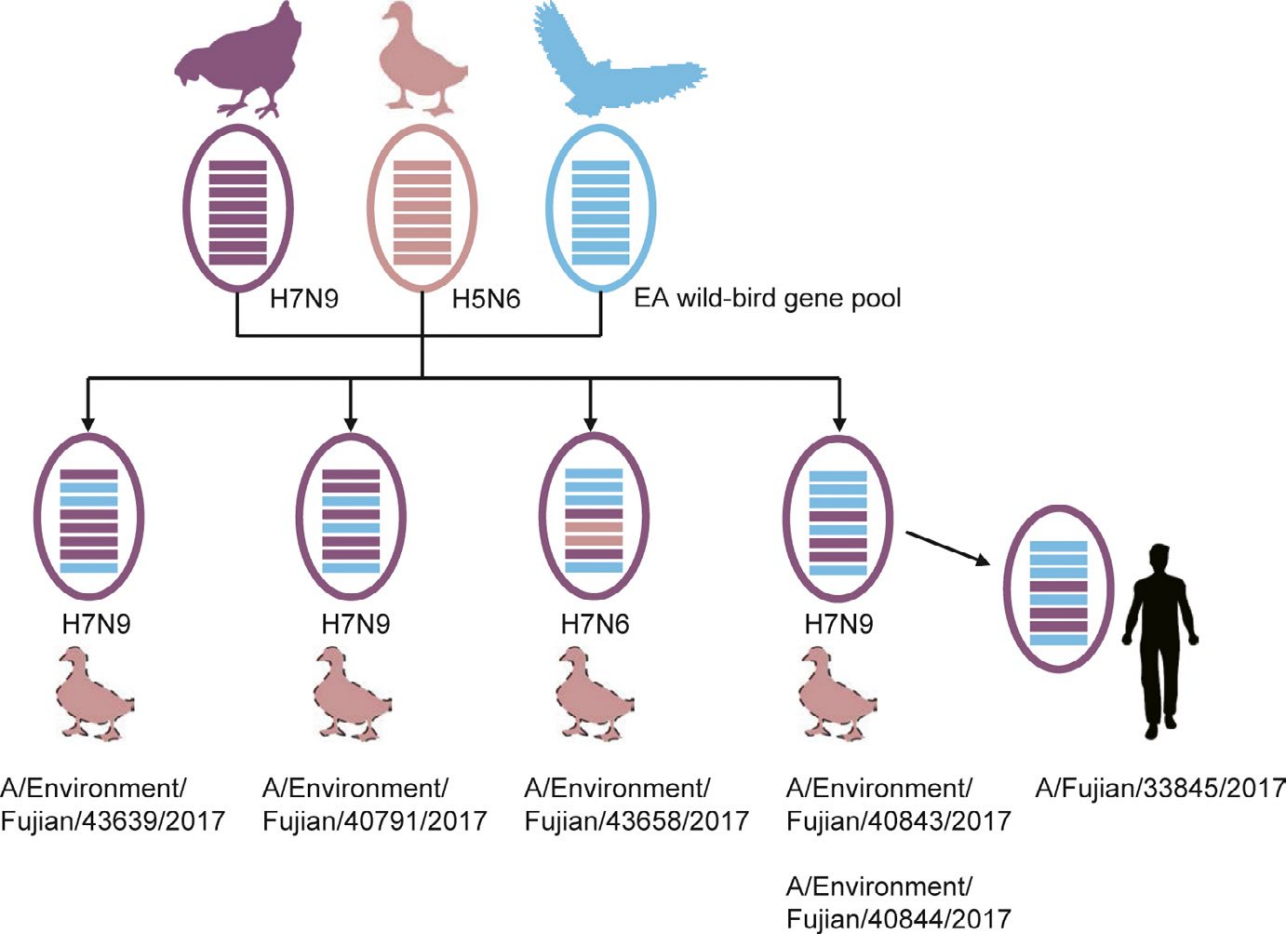
Generation of the new genotypes. The hosts are shown in cartoons and proposed hosts are in dotted stroke. The virus is demonstrated with a circle. The eight gene segments (horizontal bars), from top to bottom, include PB2, PB1, PA, HA, NP, NA, MP, and NS

According to the combination of gene sources, these HPAI H7 viruses were divided into five gene genotypes. One genotype, represented by A/Environment/Fujian/36998/2017(H7N9), was as the same as previous H7N9 viruses that contained all internal genes derived from H9N2. Four new genotypes with internal genes and NA gene from the Eurasian wild‐bird gene pool or H5N6 virus were identified (Figure 3). The new genotype that caused the human infection, represented by A/Environment/Fujian/40843/2017(H7N9), contained PB2, PB1, PA, NP, and NS genes from the Eurasian wild‐bird gene pool. Another new genotype, represented by A/Environment/Fujian/43658/2017(H7N6), contained PB2, PB1, PA, and NS genes from the Eurasian wild‐bird gene pool and NA and NP genes from H5N6 virus.

## 4 DISCUSSION

The avian influenza A(H7N9) viruses have caused five epidemic waves of human infections in China, since 2013.^18^ The fifth wave, from October 2016 to September 2017, displayed an earlier start than previous waves and a steep increase in the number of human infections.^19^ In February 2017, human infections with HPAI H7N9 were first reported in Guangdong, China. Molecular dating suggested that the HPAI H7N9 virus may emerge in the middle of 2016.^4^, ^5^ Therefore, both LPAI and HPAI H7N9 viruses were epidemic in this wave, but the LPAI H7N9 viruses were responsible for the biggest wave of human infections.^1^

In Fujian Province, all the isolates from human cases were LPAI H7N9 viruses before mid‐2017, according to the available sequences. In the present study, we reported a human case infected with an HPAI H7N9 virus in August 2017. The HPAI H7N9 viruses were also detected in poultry‐related environmental surveillance after mid‐2017. The HA genes of these HPAI H7 viruses were close relative to other HPAI H7N9 viruses, indicating that the HPAI H7 virus might have been introduced into Fujian Province from Guangdong Province where the HPAI H7N9 emerged, and Guangdong is adjacent to Fujian.

The genetic diversity of the HPAI H7N9 viruses was reportedly increased by reassortment with the LPAI H7N9 or/and H9N2 viruses.^4^, ^9^ However, five of the six HPAI H7N9 viruses detected in Fujian had acquired internal genes from the wild‐bird gene pool, not just the H7N9 or H9N2 viruses (Figures 2 and 3). One of the HPAI H7 viruses in Fujian also exchanged its NA gene with the H5N6 virus, generating a HPAI H7N6 virus. Therefore, the origins of these HPAI H7N9 variants could not be the same as those of the previous LPAI or HPAI H7N9 viruses, and the HPAI H7N9 viruses identified in this study containing wild‐bird genes could be generated in ducks rather than chickens (Figure 3).

The human isolate A/Fujian/33845/2017(H7N9) contained five internal genes from the Eurasian wild‐bird gene pool (Figures 2 and 3). The genome sequences of this human isolate shared a high similarity with one isolate from environmental surveillance, although there was no virus was isolated from the patient's duck farm. Considering that (a) the patient had no history of avian exposure except to the ducks in his farm, (b) the samples from his or his neighbor's farm were H7‐positive by real‐time RT‐PCR, and (c) the genetic relatedness to duck viruses, the source of infection could be the virus circulated in the duck farms. HPAI H7 virus in ducks was also detected in Fujian in 2017.^20^ It further supported that these HPAI H7N9 variants may generate in ducks.

The H7N9 virus poses a greater pandemic potential for its ability to recognize human receptor and cause server human disease. Until now, the viruses were circulating in poultry, and sporadic caused human infection just in Mainland China. The newly emergent genotypes of the HPAI H7 viruses raise concerns that the viruses may transmit back into wild birds, facilitating their transboundary spread, as occurred in the HPAI H5 clade 2.3.4.4 viruses, which emerged in China, spread to South Korea and Japan, and other continents by migratory birds.^21^ The new genotype viruses still carried the HA 186V residue, which indicate its dual receptor binding property. And the mammalian‐adapted mutation PB2 701N was found in the human isolates. In addition, they contained several HA1 mutations to the HPAI H7N9 candidate vaccine virus, which could affect the HA antigenicity. Since September 2017, H5/H7 bivalent vaccination was adopted as a major control strategy in poultry in Mainland China. Antigenic drift of H7N9 virus could increase the possibility of H7 vaccine escapes in the near future, which is an additional risk for re‐emergence of H7N9 virus.

Our findings suggest that the HPAI H7 virus could have derived its genetic diversity from extensive avian influenza viruses and an expanded host range, possessing a risk to public health and animal health. Although no human to human transmission was detected, considering their ability to cause severe illness in humans and domestic birds, the sustained close surveillance of HPAI H7 viruses with novel genotypes is essential.

## CONFLICT OF INTEREST

The authors declare no competing interests.
